# Enhanced Oxidative DNA-Damage in Peritoneal Dialysis Patients via the TXNIP/TRX Axis

**DOI:** 10.3390/antiox11061124

**Published:** 2022-06-06

**Authors:** Tina Oberacker, Peter Fritz, Moritz Schanz, Mark Dominik Alscher, Markus Ketteler, Severin Schricker

**Affiliations:** 1 Dr. Margarete Fischer-Bosch Institute of Clinical Pharmacology and University of Tuebingen, Auerbachstr. 112, 70376 Stuttgart, Germany; 2 Department of General Internal Medicine and Nephrology, Robert-Bosch-Hospital, Auerbachstr. 110, 70376 Stuttgart, Germany; peter.fritz2323@gmail.com (P.F.); moritz.schanz@rbk.de (M.S.); dominik.alscher@rbk.de (M.D.A.); markus.ketteler@rbk.de (M.K.); severin.schricker@rbk.de (S.S.)

**Keywords:** oxidative damage, oxidative stress, peritoneal dialysis, thioredoxin-interacting-protein, thioredoxin

## Abstract

Peritoneal dialysis (PD) is an effective method of renal replacement therapy, providing a high level of patient autonomy. Nevertheless, the long-term use of PD is limited due to deleterious effects of PD fluids to the structure and function of the peritoneal membrane leading to loss of dialysis efficacy. PD patients show excessive oxidative stress compared to controls or chronic kidney disease (CKD) patients not on dialysis. Therefore, defense systems against detrimental events play a pivotal role in the integrity of the peritoneal membrane. The thioredoxin-interacting-protein (TXNIP)/thioredoxin (TRX) system also plays a major role in maintaining the redox homeostasis. We hypothesized that the upregulation of TXNIP negatively influences TRX activity, resulting in enhanced oxidative DNA-damage in PD patients. Therefore, we collected plasma samples and human peritoneal biopsies of healthy controls and PD patients as well. Using ELISA-analysis and immunohistochemistry, we showed that PD patients had elevated TXNIP levels compared to healthy controls. Furthermore, we demonstrated that PD patients had a reduced TRX activity, thereby leading to increased oxidative DNA-damage. Hence, targeting the TXNIP/TRX system as well as the use of oxidative stress scavengers could become promising therapeutic approaches potentially applicable in clinical practice in order to sustain and improve peritoneal membrane function.

## 1. Introduction

Peritoneal dialysis (PD) is an effective method of renal replacement therapy, providing a high level of patient autonomy. In PD, the peritoneal membrane (PM) is more or less permanently in contact with glucose-based peritoneal dialysis fluids (PDFs) leading to pathophysiological changes such as peritoneal fibrosis and, at worst, encapsulating peritoneal sclerosis (EPS) mostly leading to ultrafiltration or method failure [1].

Reactive oxygen species (ROS) are generated as by-products of oxygen metabolism [2,3]. Low levels of ROS play a crucial role in many human vital physiological processes [4,5,6,7]. However, when ROS production excessively increases, important cellular structures such as protein, lipids or nucleic acids are damaged [5,6,7]. Hence, oxidative stress (OS) is involved in the onset or progression of several diseases [8,9] and plays a pivotal role in the pathogenesis and injury of the PM [1]. Therefore, maintaining the redox homeostasis by regulation of ROS formation or removal is indispensable. In this regard, cells developed different antioxidant defensive systems to overcome OS. This system comprises metallothioneins (MTs) [10], enzymatic components such as superoxide dismutase (SOD), catalase (CAT) and glutathione peroxidase (GPx) [11], or the thioredoxin (TRX)/thioredoxin-interacting-protein (TXNIP) system [12,13,14,15,16]. TXNIP expression is induced upon exposure to glucose or glucose-based PDF [17,18,19,20] or in diabetic hyperglycemia [12]. Furthermore, TXNIP binds to the catalytic active center of TRX, thereby inhibiting its reducing activity [13,21], thus resulting in a perturbation of the redox homeostasis in favor of oxidative damage. In the past, our group performed extensive studies on MTs in different tissues including the peritoneum [22,23], showing that MT expression is significantly reduced in the submesothelial layer and mesothelial cells of biopsies of patients with end-stage-renal disease (ESRD) [24]. Furthermore, our group showed that the supplementation of zinc induced MT expression in human peritoneal mesothelial cells (HPMCs) and in proximal tubular cells [25,26], and avoided tissue damage. In the present study, we aimed to analyze the TXNIP/TRX system and its impact on redox homeostasis. We hypothesized that glucose-based PDFs enhance oxidative DNA-damage in PD patients due to reduced TRX activity trigged by the upregulation of TXNIP.

## 2. Materials and Methods

### 2.1. Patients and Patient’s Material

All peritoneal biopsies were obtained from the peritoneal biopsy registry at the Robert-Bosch-Hospital, Stuttgart, Germany. Sample collection was carried out according to the Declaration of Helsinki and was approved by the ethics committee of the Eberhard-Karls University Tuebingen, Germany (process number: 322/2009BO1 and 609/2019BO2). All patients had given their written informed consent regarding a scientific workup of blood samples and tissues taken during routine surgery.

Blood samples were collected 1 day prior to surgery and stored at −20 °C. Peritoneal biopsies were taken from control patients during the following surgeries: hemicolectomy, inguinal hernia, cholecystectomy, or ileostomy displacement. Biopsies prior to PD initiation (uremic controls) were collected during catheter implantation whereas biopsies from PD patients were taken at the time of catheter removal, correction of a catheter malposition or as diagnostic biopsies for suspected EPS. Biopsies were obtained by the suture technique as described previously [27], incubated in RNAlater or pre-chilled in liquid nitrogen and stored at −80 °C. Patients’ baseline characteristics are shown in Table 1.

### 2.2. Thioredoxin Activity Assay

Peritoneal biopsy lysates were prepared as previously described [28]. TRX activity of 20 µg protein samples was analyzed using the fluorescence-based assay kit (FkTRX-04, BIOZOL, Eching, Germany) according to the manufacturer’s instructions. The increase in fluorescence was measured using a multimode microplate reader (Enspire PerkingElmer, Waltham, MA, USA) and displayed as % TRX activity compared to control.

### 2.3. Enzyme-Linked Immuno-Sorbent Assay (ELISA) 

The TXNIP ELISA kit (ABIN2951760; antikörper-online) and the DNA Damage Competitive ELISA Kit (EIADNAD, Thermofisher Scientific, Karlsruhe, Germany) were used according to the manufacturer’s instructions. Absorbance was measured using a multimode microplate reader (Enspire PerkingElmer). 

### 2.4. Immunohistochemistry (IHC)

Peritoneal biopsies were formalin-fixed in 4% buffered formalin and paraffin-embedded following routine protocols [29]. Immunohistochemistry was performed as previously described [28,30,31]. Used antibodies: anti-TXNIP (ab188865; 1:200, Abcam, Cambridge, UK), anti-TRX (sc-271281; 1:100, Santa Cruz), anti- γH2AX (9718S; 1:100, CST). 

For morphology, slides underwent hematoxylin and eosin (HE) staining. Tissue sections were examined with Olympus VS120 automated slide scanner equipped with a BX61VS microscope (objective: UPLSAPO 20× or UPLSAPO 2 40×, Olympus, Tokyo, Japan). Mesothelial cell loss was evaluated as: completely contained (0); partially lost (1); completely lost (2). Mean submesothelial thickness was evaluated by measuring the thinnest and thickest layer using the imaging software (OlyVIA V3.3, Olympus). 

### 2.5. Evaluation of Histo-Score

Immunohistochemistry results were semi-quantitatively evaluated as previously described [28,32]. The following structures or cells of these structures were analyzed: mesothelial structure, submesothelial structure, adipocytes and vessels. The percentage of stained cells or structures (<10% (0), 10–25% (1), 25–50% (2), 50–75% (3), ≥75% (4)) and staining intensity (no staining (0), weak (1), moderate (2) or strong (3)) were determined and the final score was calculated by the formula:

Histo-Score = (percentage mesothelial structure × staining intensity mesothelial structure) + (percentage submesothelial structure × staining intensity submesothelial structure) + (percentage adipocytes × staining intensity adipocytes) + (percentage vessels × staining intensity vessels) 

### 2.6. Statistical Analysis

For comparison of the subgroups, a Kruskal-Wallis test and Dunn’s post-hoc analysis was performed using GraphPad Prism 9 (GraphPad Software Inc., San Diego, CA, USA).

## 3. Results

To test our hypothesis that glucose-based PDFs enhance oxidative DNA-damage in PD patients due to reduced TRX activity trigged by TXNIP upregulation, we collected plasma samples and human peritoneal biopsies from our cohort. Since TXNIP is also upregulated upon aging [7] and in a high glucose environment [17,33], we used widely age-matched samples and excluded diabetic patients from our study. Furthermore, rat studies showed that hypertension could also influence TXNIP expression [34]. However, we could not exclude hypertensive CKD patients from our cohort since the majority of CDK patients also suffer from hypertension (Table 1).

### 3.1. TXNIP Is Upregulated in PD Patients

**Figure 1:**
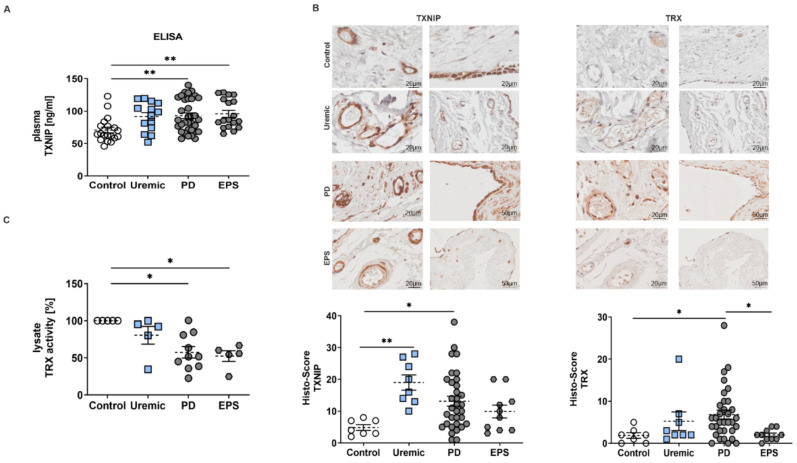
TXNIP is upregulated in PD patients. (**A**) TXNIP expression was analyzed by ELISA using plasma samples (mean ± S.E.M.; **: *p* < 0.01; control: *n* = 19, uremic: *n* = 14, PD: *n* = 34 and EPS: *n* = 18). (**B**) Representative peritoneal sections showing vessels (first row) and mesothelium (second row) stained for TXNIP and TRX. Scatter blots show the Histo-Score of the sections (mean ± S.E.M.; **: *p* < 0.01, *: *p* < 0.05; control: *n* = 7, uremic: *n* = 8, PD: *n* = 33 and EPS: *n* = 10). (**C**) TRX activity of biopsy lysates was normalized to controls (mean ± S.E.M.; *: *p* < 0.05; *n* = 5 except for PD: *n* = 10).

We observed that plasma samples of PD or EPS patients showed significantly higher TXNIP concentrations than controls and predialysis patients (Figure 1A). An analysis of biopsy sections demonstrated that TXNIP and TRX were mainly but not exclusively expressed in mesothelial cells and endothelial cells of the vessel walls of the peritoneal membrane (Figure 1B). Semi-quantitative scoring of IHC staining showed that TXNIP is upregulated in predialysis and PD patients. However, we do not observe an increase in TXNIP in biopsy samples of EPS patients as shown by ELISA (compare Figure 1A,B). The evaluation of TRX by Histo-Score showed only a significant increase in PD patients compared to healthy controls (Figure 1B). However, mRNA expression levels of other redox-related genes such as superoxide-dismutase (SOD) and catalase were not changed (Figure S1). Since TRX is negatively regulated by TXNIP [21], we investigated TRX activity in biopsy lysates. We observed a significant reduction in TRX activity of PD and EPS patients compared to controls (Figure 1C), although TRX expression seemed to be upregulated in biopsy sections (see Figure 1B).

### 3.2. Pathological Changes in the Peritoneal Membrane of PD Patients

**Figure 2:**
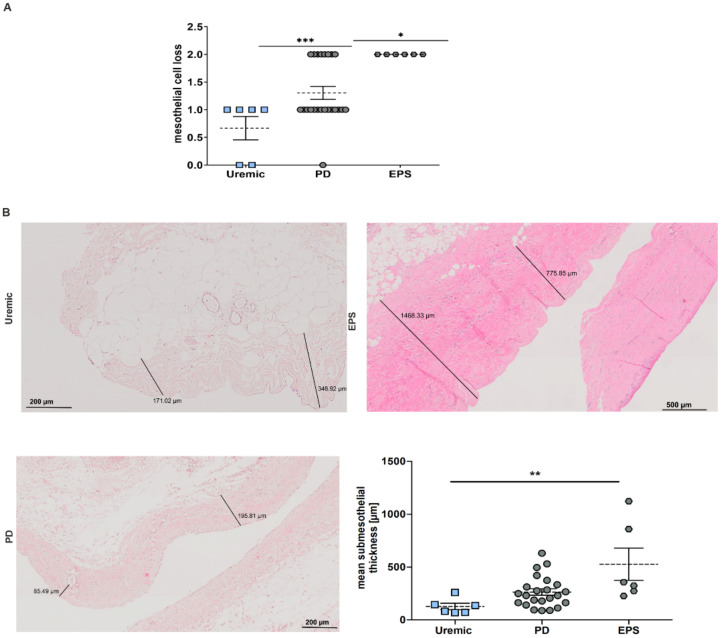
Pathological changes in the peritoneal membrane of PD patients. (**A**) Scatter blot of mesothelial cell loss of peritoneal biopsies (mean ± S.E.M.; *: *p* < 0.05, ***: *p* < 0.001; *n* = 6 except for PD: *n* = 23). (**B**) Representative HE sections of biopsy sections. Scatter blot shows the mean submesothelial thickness (mean ± S.E.M.; **: *p* < 0.01; *n* = 6 except for PD: *n* = 23).

Changes in the peritoneal membrane during PD over time were analyzed for mesothelial cell loss and increase in the submesothelial compact zone. We observed a significant increase in mesothelial cell loss in PD and EPS patients compared to predialysis patients (Figure 2A) and an increase in fibrosis between uremic and EPS patients measuring submesothelial thickness (Figure 2B).

### 3.3. Enhanced Oxidative DNA-Damage in Plasma and the Peritoneal Membrane of PD Patients

**Figure 3:**
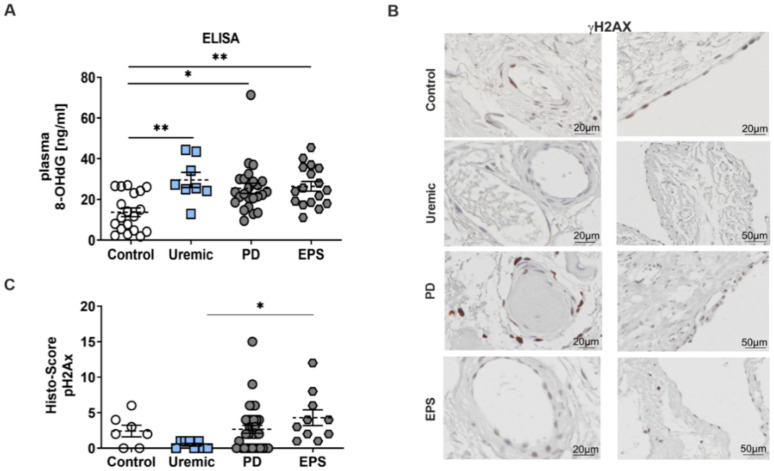
Enhanced oxidative DNA-damage in plasma and the peritoneal membrane of PD patients. (**A**) 8-OHdG content was analyzed by ELISA using plasma samples (mean ± S.E.M.; *: *p* < 0.05; **: *p* < 0.01; control: *n* = 19, uremic: *n* = 8, PD: *n* = 24 and EPS: *n* = 17). (**B**) Representative peritoneal sections showing vessels (first row) and mesothelium (second row) stained for γH2AX. (**C**) Scatter blots show the Histo-Score of the sections (mean ± S.E.M.; *: *p* < 0.05; control: *n* = 7, uremic: *n* = 8, PD: *n* = 33 and EPS: *n* = 10).

Reduced TRX activity induces a perturbation of the redox equilibrium in favor of oxidative damage. Therefore, we evaluated the level of oxidative DNA-damage using 8-hydroxy-2′-deoxyguanosine (8-OHdG) and γH2Ax phosphorylation as biomarkers. We observed a significant increase of 8-OHdG concentration patients’ plasma compared to controls (Figure 3A). We also observed an induction of γH2Ax in mesothelial cells and in the peri-capillary walls as well. This increase in oxidative DNA-damage is only significant between predialysis and EPS patients (Figure 3B,C).

## 4. Discussion

Long-term exposure to glucose-based dialysate leads to pathological changes in the PM such as peritoneal fibrosis, ultrafiltration failure and mesothelial cell loss [27,35]. Here, we present data that TXNIP is upregulated during peritoneal dialysis resulting in reduced TRX enzyme activity in membrane lysates of PD and EPS patients due to less free TRX. This change in the redox equilibrium was associated with an increase in oxidative DNA-damage in the plasma and especially in the peritoneal membrane of patients, which could lead to changes in the pathophysiology of the membrane potentially resulting in method failure.

In general, we observed differences between TXNIP expression analyzed by ELISA or by semi-quantitative evaluation of the Histo-Score especially in the EPS subgroup (compare Figure 1A,B). This discrepancy could be explained by the fact that TXNIP expression in biopsy samples was mainly observed in mesothelial cells and the vessel walls. However, EPS biopsies showed an increase in mesothelial cell loss, as shown by Figure 2A. Therefore, we especially evaluated the expression in the submesothelial zone, in vessel walls and adipocytes. 

In principle, TXNIP expression negatively influences TRX activity as except for pre-dialysis patients. Here, no change in TRX activity compared to controls could be observed, although the expression of TXNIP was significantly increased (Figure 1A–C). We assumed that the cellular composition, for example, the number of mesothelial cells or the presence of vessels differs in the lysates compared to the paraffin sections.

To the best of our knowledge, this is the first study investigating the effects of peritoneal dialysis on TXNIP expression and its impact on redox homeostasis in human peritoneal biopsy samples. In accordance with our findings are several studies in rats or on primary mesothelial cells showing increased TXNIP expression upon exposure to high glucose-based PDFs [20]. In general, redox homeostasis is critical for cell viability, activation, proliferation, and organ function [36,37]. Further, the accumulation of ROS is associated with the pathogenesis of CKD [38,39,40,41] and influences cellular function and mortality in PD patients [1,42,43,44,45]. Our results showing increased oxidative DNA-damage are consistent with previous studies demonstrating increased damage due to attenuated defense mechanisms [7,46] and studies showing that both hemodialysis and peritoneal dialysis induces oxidative DNA-damage in patients or in peritoneal mesothelial cells of PD patients [44,47,48,49]. In addition, previous studies from our group demonstrated that ESRD patients showed reduced expression of MT presuming a disbalance in redox homeostasis leading to increased oxidative damage [24,35]. Beyond, the increase in γH2Ax in mesothelial cells and in the peri-capillary of peritoneal biopsy sections are in accordance with previous studies from our group, showing that unphysiological PDFs could induce apoptosis of mesothelial cells [35]. In our study, we could not completely exclude that age [7] or hypertension of CDK patients may affect oxidative stress balance as well. However, we widely analyzed age-matched patients of the different subgroups. Furthermore, we could not entirely exclude that higher glucose in PDFs may possess a relatively more significant impact on membrane damage than PD over time.

To date, several studies have shown that targeting the TRX system [50] or the use of antioxidative substances offer novel therapeutic opportunities for the treatment of several diseases [51,52,53,54,55,56,57]. So far, no effective drugs affecting oxidative stress are used in clinical practice. Promising results were obtained by our and other groups, showing that zinc supplementation induces MT expression in vitro and thereby protecting against oxidative damage [25,26]. Beyond, astaxanthin supplementation prevented peritoneal fibrosis in rats [53] and resveratrol treatment improved ultrafiltration in PD patients [54,55]. Interestingly, the SGLT-2 inhibitor empagliflozin ameliorated high-glucose induced apoptosis and reduced TXNIP expression in mouse mesangial cells [56]. Based on our findings, it could be speculated that such antioxidant treatments or even the use of SGLT-2 inhibitors could be also beneficial in peritoneal dialysis patients.

## 5. Conclusions

In summary, we elucidated the hypothesis that the glucose-dependent upregulation of TXNIP induces a perturbation of the intracellular redox equilibrium, favoring oxidative stress and potentially leading to pathological changes in the peritoneum. Therefore, the manipulation of the redox equilibrium may also be a sustainable approach for therapy in PD patients, potentially delaying functional deterioration of the peritoneal membrane.

## Figures and Tables

**Table 1:** Clinical data of study patients.

Number (*n*)	Control 19	Uremic 18	PD 51	EPS 19
Age (years), median (IQR)	52.0 (32.0–65.0)	66.0 (48.5–71.5)	51.0 (44.0–67.0)	52.0 (45.0–59.0)
Female/male (*n*)	14/5	6/12	16/35	4/15
PD-duration (months)				
median (IQR)			22.0 (11.0–45.0)	72.0 (53.0–104.0)
Composition of PD-Fluid				
Neutral pH (*n*)			20	9
Acidic pH (*n*)			23	6
Both or N.D. (*n*)			8	4
Icodextrin (*n*)			23	15
Transporter status				
High (*n*)			5	6
Average (*n*)			16	9
Low (*n*)			10	3
N.D. (*n*)			20	1
Diabetes	excluded	excluded	excluded	excluded
Hypertension (*n*,%)	0 (0%)	17 (94%)	43 (84%)	16 (84%)
Smoking status (*n*,%)	1 (5%)	1 (6%)	8 (16%)	2 (11%)
Laboratory				
Haemoglobin (g/L), median (IQR)	136.5 (124.0–151.8)	106.0 (92.0–112.0)	114.0 (106.8–126)	106.0 (86.0–118.3)
N.D. (*n*)	9		5	1
Leucocytes (109/L), median (IQR)	5.7 (5.0–7.1)	5.8 (5.1–6.5)	7.1 (5.6–7.1)	6.7 (5.1–8.8)
N.D. (*n*)	9		4	1
Phosphate (mmol/L), median (IQR)		1.8 (1.3–2.1)	1.4 (1.1–1.8)	1.5 (1.1–1.7)
N.D. (*n*)			7	3
Calcium (mmol/L), median (IQR)	2.3 (2.2–2.4)	2.1 (2.0–2.2)	2.3 (2.2–2.5)	2.3 (2.1–2.4)
N.D. (*n*)	9		5	1
PTH (pmol/L), median (IQR)		28.4 (20.6–32.3)	25.1 (12.0–31.0)	18.7 (4.5–78.2)
N.D. (*n*)		5	15	5
Urea (mg/dL), median (IQR)		152.0 (121.5–185.5)	96.5 (62.8–132.0)	101.0 (67.3–113.3)
N.D. (*n*)			7	1
Creatinine (mg/dL), median (IQR)	0.8 (0.7–1.0)	6.1 (5.2–7.0)	6.7 (4.1–9.9)	6.8 (5.6–8.9)
N.D. (*n*)	9		4	1

IQR: interquartile range, EPS: encapsulating peritoneal sclerosis, *n* = number of values, N.D. not determined, PD: peritoneal dialysis. PTH: parathyroid hormone; Percentages rounded to whole numbers.

## Data Availability

The data presented in this study are available on request from the corresponding author. The data are not publicly available due to ethical restrictions.

## Supplementary Materials

The following supporting information can be downloaded at: https://www.mdpi.com/article/10.3390/antiox11061124/s1, Figure S1: Gene expression of redox-related genes is not elevated in peritoneal dialysis patients.
