# Supplementary material for: Enhanced Oxidative DNA-Damage in Peritoneal Dialysis Patients via the TXNIP/TRX Axis
Source: Antioxidants (Basel). 2022 Jun 6;11(6):1124. doi: 10.3390/antiox11061124 (PMC9220040; doi:10.3390/antiox11061124)
Supplement: Supplementary file 1 [file antioxidants-11-01124-s001.zip › antioxidants-1747463-supplementary.pdf]

Supplementary Materials:

Tissue homogenization

For RNA-isolation 50-100 mg peritoneal biopsy material was shredded in 600 µl lysis buffer (mirVana™ miRNA Isolation Kit, Thermo Fisher Scientific, Karlsruhe, Germany) with an ulta-turrax (16,000× *g*, 1 min). Thereafter, the supernatant was transferred to tubes filled with ceramic balls and homogenized using the FastPrep system (3 times, 6 m/s, 20 sec.; MP Biomedicals, Eschwege, Germany). Last, the supernatant was centrifuged at 16,000× *g* for 1 min.

Quantitative PCR (qPCR)

Total RNA was isolated with the mirVana™ miRNA Isolation Kit (Thermo Fisher Scientific, Karlsruhe, Germany) according to the manufacturer's instructions. Total RNA (1 µg) was reverse transcribed with an RT-PCR kit (Applied Biosystems, Darmstadt, Germany) and afterwards diluted 1:1 with water. qPCR was performed using Power SYBR Green PCR Master Mix (Applied Biosystems). Gene expression was analyzed using the 7500 Real-Time PCR Systems and Sequence Detection Software, version 2.3 (Applied Biosystems). Gene expression was evaluated using the ΔΔCT method and GAPDH as reference gene. The following primer sequences were used:
NM_001752.4 (*Catalase)*: forward 5'-3' GCC TGG GAC CCA ATT ATC TT, reverse 5'-3' GAA TCT CCG CAC TTC TCC AG; NM_002046.7 (*GAPDH)*: forward 5'-3' GCA TCT TCT TTT GCG TCG, reverse 5'-3' TGT AAA CCA TGT AGT TGA GGT; NM_000454.5 (*SOD1)*: forward 5'-3' CAA TGT GAC TGC TGA CA AAG, reverse 5'-3' GTG CGG CCA ATG ATG CAA T; NM_000636.4 (*SOD2)*: forward 5'-3' GAC AAA CCT CAG CCC TAA CG, reverse 5'-3' GAA ACC AAG CCA ACC CCA AC.

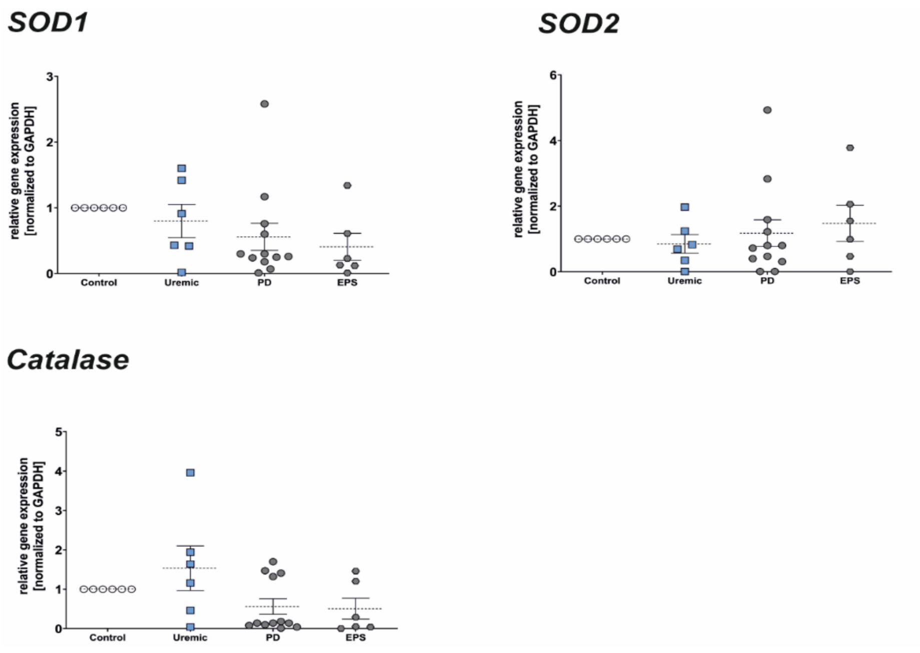

Figure S1. ***Gene expression of redox-related genes is not elevated in peritoneal dialysis patients.***

mRNA expression of SOD1, SOD2 and catalase was determined by qPCR. Gene expression was normalized to GAPDH as reference gene. Statistical differences were determined by a by a Kruskal-Wallis test and Dunn's post hoc analysis (mean ± .S.E.M.; n = 6).
